# Co-delivery of glycyrrhizin and doxorubicin by alginate nanogel particles attenuates the activation of macrophage and enhances the therapeutic efficacy for hepatocellular carcinoma

**DOI:** 10.7150/thno.35972

**Published:** 2019-08-14

**Authors:** Qiang-Song Wang, Li-Na Gao, Xiao-Ning Zhu, Yan Zhang, Chuang-Nian Zhang, Dong Xu, Yuan-Lu Cui

**Affiliations:** 1 Tianjin Key Laboratory of Biomedical Materials, Institute of Biomedical Engineering, Chinese Academy of Medical Science & Peking Union Medical College, Tianjin 300192, PR China; 2 Tianjin State Key Laboratory of Modern Chinese Medicine, Research Center of Traditional Chinese Medicine, Tianjin University of Traditional Chinese Medicine, Tianjin, 301617, PR China; 3 College of Pharmacy, Jining Medical University, Rizhao, PR China

**Keywords:** Glycyrrhizin, Alginate, Nanogel particles, Macrophage, Combination therapy

## Abstract

Nanocarrier drug delivery systems (NDDS) have been paid more attention over conventional drug delivery system for cancer therapy. However, the efficacy is hampered by the fast clearance of activated macrophage from the blood circulation system. In this study, glycyrrhizin (GL) was introduced into alginate (ALG) nanogel particles (NGPs) to construct multifunctional delivery vehicle to decrease the fast clearance of activated macrophage and enhance the anticancer efficacy with the combination therapy of GL and doxorubicin (DOX).

**Methods**: We firstly synthesized the GL-ALG NGPs with intermolecular hydrogen bond and ionic bond as the multifunctional delivery vehicle. The immune response and phagocytosis of macrophage on GL-ALG NGPs were investigated on RAW 264.7 macrophages. The pharmacokinetic study of DOX loaded in GL-ALG NGPs was performed in rats. The active targeting effects of GL-ALG NGPs were further studied on hepatocellular carcinoma cell (HepG2) and H22 tumor-bearing mice. Moreover, the anticancer molecular mechanism of DOX/GL-ALG NGPs was investigated on HepG2 cells *in vitro* and tumor-bearing mice *in vivo*.

**Results**: GL-ALG NGPs could not only avoid triggering the immuno-inflammatory responses of macrophages but also decreasing the phagocytosis of macrophage. The bioavailability of DOX was increased about 13.2 times by DOX/GL-ALG NGPs than free DOX in blood. The mice with normal immune functions used in constructing the tumor-bearing mice instead of the nude mouse also indicated the good biocompatibility of NGPs. GL-mediated ALG NGPs exhibited excellent hepatocellular carcinoma targeting effect *in vitro* and *in vivo*. The results suggested that the anticancer molecular mechanism of the combination therapy of glycyrrhizin and doxorubicin in ALG NGPs was performed via regulating apoptosis pathway of Bax/Bcl-2 ratio and caspase-3 activity, which was also verified in H22 tumor-bearing mice.

**Conclusion**: DOX/GL-ALG NGPs could attenuate the activation of macrophage and enhance the therapeutic efficacy for hepatocellular carcinoma. Our results suggest that the combination therapy would provide a new strategy for liver cancer treatment.

## Introduction

Liver cancer is the second leading cause of cancer mortality worldwide, causing more than 700,000 deaths annually 1. Hepatocellular carcinoma (HCC), the most common histological type of liver cancer, accounts for 90% of all cases of primary liver cancer, with nearly 800, 000 new cases annually 2. Liver transplantation and chemotherapy are the main treatments for liver cancer, but the unavailability of liver donors is a major issue when considering surgery. The therapeutic efficacy of present cancer chemotherapies is limited by the narrow therapeutic indexes due to high non-specific toxicity for normal tissues or organs 3. Chemotherapy drugs are usually non-specific small molecules that have poor pharmacokinetic profiles and simply distribute freely throughout the body, leading to diminishing their potency at actual target sites. Therefore, the development of a delivery system to alter chemotherapeutic drug distribution* in vivo*, enhance its deposition in the tumor sites and reduce its side-effect are required for the specific treatment of cancer.

Nanocarrier drug delivery systems (NDDS), such as nanoparticles (NPs), have been paid more attention over the last two decades to overcome the limitations of conventional drug delivery system for cancer therapy 4. NDDS provide advantages over the administration of free drugs, including targeting the ability to increase accumulation at tumor sites 5, enhancing the intracellular concentration of drugs in cancer cells while avoiding toxicity to normal cells 6, and realizing controlled and sustained release to enhance drug bioavailability 7. However, the nanoparticles are also considered as an intruder by the innate immunity system, which can be recognized and engulfed by macrophages. In this case, the nanoparticles will be removed from the blood circulation system and lose their function quickly, leading to a dramatic reduction in efficiency of diagnostics and therapeutics. To escape from the reticular-endothelial system (RES), like macrophage cells for longer circulation times, the nanoparticles are usually coated with a layer of the hydrophilic and biocompatible polymer 8.

Natural chemicals have gained many attentions in cancer therapy due to the safety profile (low toxicities). Unfortunately, the antitumor effect of natural products alone is usually far from satisfactory. Glycyrrhizin (GL, **Figure S1A**), is the main active component in licorice with multiple biochemical and pharmacological activities, including antiviral, immunoregulatory, anti-inflammatory, etc 9-11. Recent studies indicated the cardioprotective effect and high anticancer efficacy in hepatocellular carcinoma 12, 13. Glycyrrhetinic acid (GA, **Figure S1B**), the aglycone of GL, is also an active component of licorice. The previous study showed that there were specific binding sites for GL and GA on the cellular membrane of rat hepatocytes 14. Growing interest has focused on GL or GA-mediated liver targeted drug delivery systems. GL-modified chitosan nanoparticles showed preferentially accumulation in hepatocytes *via* a ligand-receptor interaction 15. Our previous studies indicated that GA-modified nanoparticles could perform the liver targeting effect and inhibit the tumor growth against the liver 16, 17. Although GL or GA by polymer-drug conjugate to construct the targeting delivery system showed active liver targeting effect, the immunoregulatory and anti-inflammatory effects of GL or GA were decreased dramatically by steric hindrance effect. Moreover, our recent study showed that the injectable GL-ALG hydrogels existed good biological activity for three-dimensional cell culture in liver tissue engineering 18.

**Scheme 1:**
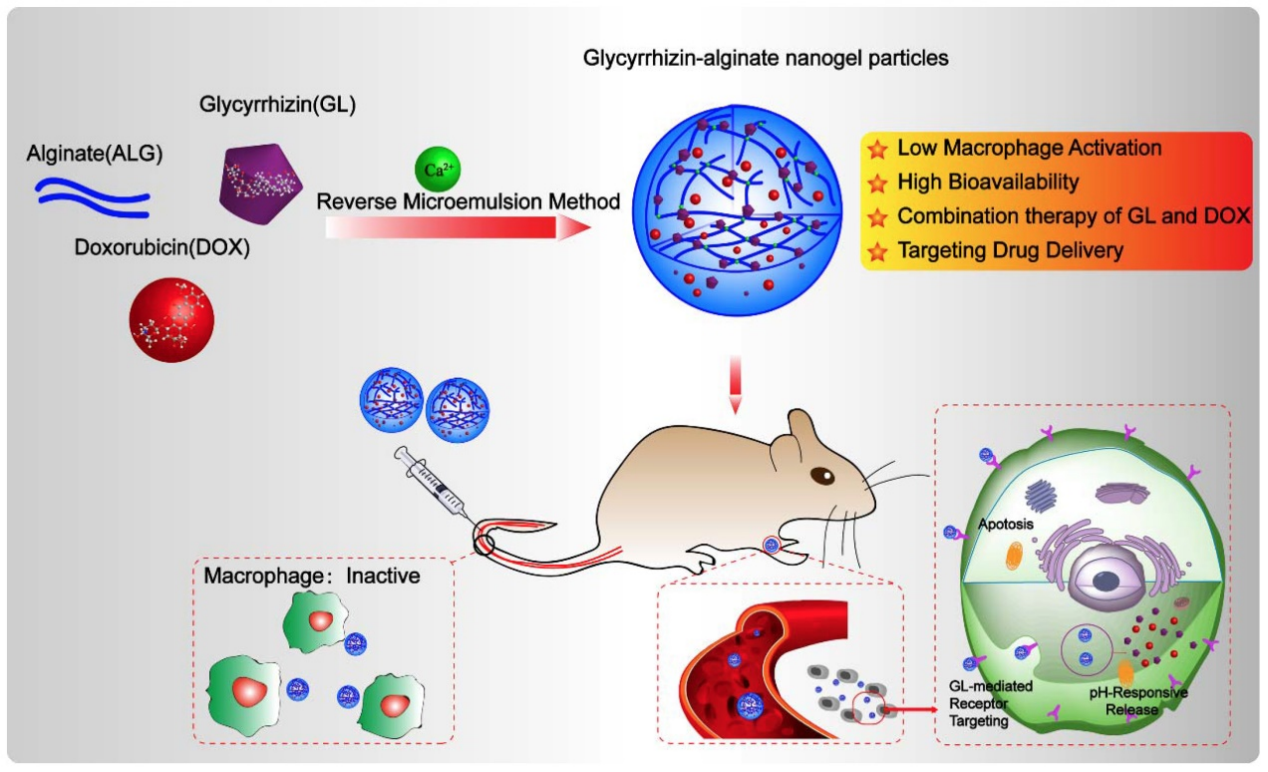
The schematic illustration of alginate nanogel particles co-delivering glycyrrhizin and doxorubicin to attenuate the activation of macrophage and enhance the therapeutic efficacy for hepatocellular carcinoma.

In order to avoid being recognized and engulfed by the macrophage cells, and preserve the immunoregulatory effect of GL, in this study, GL was introduced in ALG NGPs by intermolecular hydrogen bond which could deliver DOX for the combination therapy to suppress hepatic tumor growth in a subcutaneous mouse model of liver cancer. The GL-ALG NGPs is given more prospects (**Scheme 1**): (i) Reducing the phagocytosis of macrophages on NGPs with the immunoregulatory activity of GL. (ii) Increasing the bioavailability of DOX and delivering DOX to the specific binding sites on the cellular membrane of hepatocytes. (iii) The combination therapy of DOX and GL could enhance anti-cancer efficiency and reduce the cardiotoxicity of free DOX. In addition, we investigated the underlying molecular mechanisms on anti-tumor effects of combination therapy.

## Materials and Methods

### Materials

Sodium alginate (viscosity: 160 mPa s), the rhodamin B isothiocyanate (RBITC) were provided by Sigma-Aldrich Co. (St. Louis, MO, USA). Glycyrrhizin (HPLC purity > 99%) was purchased from Yongheng Biotech, Co., Ltd. (Shanghai, China). Doxorubicin hydrochloride (HPLC purity > 98%) was obtained from Wuhan Yuancheng Gongchuang Technology Co., Ltd. (Wuhan, China). Mammalian cell lysis kit, UNIQ-10 column Trizol total RNA extraction kit, anti-β-actin, anti-Bax, anti-Bcl-2, anti-Bcl-XL antibodies, and peroxidase-conjugated secondary antibody were bought from Sangon Biotech, Co., Ltd. (Shanghai, China). Improm-II Reverse Transcription System was purchased from Promega Corporation (Madison, Wisconsin, USA). FastStart Universal SYBR Green Master (ROX) kit was purchased from Roche (Mannheim, Germany). Acetonitrile and methanol were of chromatographic grade. All the other chemicals were of analytical grade.

Human hepatocellular carcinoma (HepG2) cells were maintained in Dulbecco′s Modified Eagle Medium (DMEM) supplemented with 10% fetal bovine serum (FBS) at 37 °C in a humidified incubator containing 5% CO_2_. Kunming mice (18-22 g) were housed in a ventilated, temperature-controlled and standardized sterile animal room. The animal experiment procedures were performed according to the National Institutes of Health Guide for Care and Use of Laboratory Animals, and the protocol was approved by the Animal Ethics Committee of Chinese Academy of Medical Science & Peking Union Medical College.

### Preparation and characterization of DOX/GL-ALG NGPs

DOX/GL-ALG NGPs were synthesized with the phase inversion temperature (PIT) emulsification method reported previously with some modification. In brief, sodium alginate solution (0.5%, w/v) was obtained by dissolving the sodium alginate in deionized water with magnetism stirring for 6 h at 45 °C. Paraffin oil (150 ml) containing 1% of Span-80 and 0.3% of Tween 80 was mixed for 30 min under stirring of 500 rpm, which was stirred another 30 min after adding 2 ml doxorubicin hydrochloride solution (20 mg/ml). An amount of 45 ml sodium alginate solution was dropped into 150 ml paraffin oil stirring for 60 min to form uniform-sized droplets, and 15 ml CaCl_2_ solution (0.1%, w/v) containing 1% of glycyrrhizin was added and stirred to gel for 1 h. The DOX/ GL-ALG NGPs were collected by centrifugation and lyophilized for 24 h.

The morphological examination was performed with transmission electron microscopy (TEM, H-7650, Hitachi, Japan). FT-IR spectra of samples were recorded using a Thermo Nicolet iS10 FT-IR spectroscopy (Thermo Scientific, MA, USA) in the wave number range 4000-400 cm^-1^ using KBr pellets. Differential scanning calorimetry (DSC) curves were recorded using a differential scanning calorimeter (Jade DSC, Perkin-Elmer Corp, Wilton, USA). X-ray diffraction patterns of dried samples were analyzed by an XRD analyzer (D/Max-2500, Rigaku D, Tokyo, Japan) with Cu-Kα radiation (λ = 0.154 nm) generated at 40 kV and 100 mA as the X-ray source. The intensity was recorded in the 2θ range of 3 to 50 ^o^. The particle size and the zeta potential of nanogel particles were determined by dynamic light scattering (DLS) and laser Doppler anemometry using a Zetasizer 3000 (Malvern Instruments, UK) at room temperature, and each group was analyzed in triplicate. The interaction of GL, ALG, and Ca^2+^ was also investigated with different concentrations of three components. The viscoelastic behavior of different combinations was performed with a AR 2000 ex Rheometer (TA Instrument, New Castle, DE, USA), and the change of conformational (secondary) structure was performed at room temperature on Jasco J-715 spectrophotometer (Jasco Corp., Tokyo, Japan).

### The drug *in vitro* release studies

The DOX loaded in GL-ALG NGPs were separated with ultrafiltration tube with a 50 kDa cut off (Millipore Corporation, USA). The amount of free DOX in the filtrate was measured by HPLC equipped with a Kromasil 100-5 C18 column following the method of Chinese pharmacopeia 2015 edition. The* in vitro* release profiles of DOX from nanogel particles were evaluated using the following procedures: Two milliliters of nanogel particles suspension were transferred to a dialysis membrane bag with a molecular cutoff of 7 kDa, and the bag was immersed into 40.0 ml of PBS (pH=5, 7.4) incubated at 37 °C. At appropriate intervals, 3.0 ml of the release medium were removed and replaced by 3.0 ml fresh medium. The amount of DOX in the release medium was evaluated by HPLC with the above method.

### The immune response of macrophage on nanogel particles

The murine macrophage cell line (RAW 264.7 cells) was used to investigate the immune response of macrophage against the nanogel particles 19. RAW 264.7 cells were treated with LPS (0.2 μg/ml) as a positive control, various concentrations of GL-ALG NGPs (84, 21, 5.25 μg/ml calculated by the weight of NGPs) without LPS for 24 h. Atto 565 Phalloidin, DAPI was used to stain the cytoskeleton and cell nuclei, respectively, and DAF-FM diacetate was used to evaluate intracellular NO production, which was evaluated by using CLSM. The nitrite accumulated in culture medium was measured as an indicator of NO production using Griess reagent system (Promega, USA). The secretions of IL-6 and TNF-α from the cells were detected using mouse ELISA kits (Invitrogen, Carlsbad, CA). The cell morphologies were also investigated using scanning electron microscopy (SEM, Quanta 200, FEI, USA).

The cellular uptake of RAW264.7 cells on ALG NGPs and GL-ALG NGPs was evaluated using RBITC labeled alginate of nanogel particles in accordance with the protocol of Davis et al 20. A quantitative determination of cellular phagocytosis on NGPs was performed by flow cytometry as follows. Briefly, RAW264.7 cells were incubated with RBITC-ALG NGPs or RBITC-GL-ALG NGPs at 37 °C for 2, 4, 6, and 8 h, respectively. Then, cells were harvested and suspended in 0.5 ml of PBS containing 1% paraformaldehyde (PFA), and flow cytometry analysis was performed with a FACS Aria Cell Sorter (Becton Dickinson). All data of the mean fluorescence signal were obtained from a population of 10 000 cells. The samples were detected by flow cytometry for RBITC with the excitation wavelength and emission wavelength at 488 and 575 nm, respectively.

### Pharmacokinetics study of DOX in nanogel particles *in vivo*

Male Sprague-Dawley (SD) rats were intravenous administered DOX or DOX/GL-ALG NGPs at the dose of 2.5 mg/kg (calculated by free DOX dose) body weight. At each time point (5, 10, 15, 30, 60, 120, 240, 360, 480, 720, and 1440 min), blood (0.4 ml) was collected from the retro-orbital plexus of the rats under anesthesia and transferred to a heparin-pretreated centrifuge tube. Then, the samples were centrifuged at 3500 rpm for 15 min to collect plasma and stored at -80 °C for further analysis. The DOX concentration was determined according to the published literature using HPLC analysis with a fluorescence detector 21.

### Cellular uptake and viability of hepatocellular carcinoma cell on nanogel particles

HepG2 cells were treated with RBITC-ALG NGPs or RBITC-GL-ALG NGPs at 4, 37 °C, respectively, for 4 h. Then the cells were incubated with DAPI solution and DiO solution for another 10 min. The samples were analyzed with three fluorescent dyes by confocal laser scanning microscope (CLSM, Carl Zeiss, LSM 710, Germany). The RBITC was used to observe the NGPs in cells. The DAPI and DiO were performed to observe the cell nucleus and cell membrane, respectively. A quantitative determination of cellular phagocytosis on NGPs was performed by flow cytometry as follows. Briefly, HepG2 cells were incubated with RBITC-ALG NGPs and RBITC-GL-ALG NGPs at 4, 37 °C for 2, 4, 6, and 8 h, respectively. Then, cells were harvested and suspended in 0.5 ml of PBS containing 1% paraformaldehyde (PFA), and flow cytometry analysis was performed with a FACS Aria Cell Sorter (Becton Dickinson). All data of the mean fluorescence signal were obtained from a population of 10 000 cells. The samples were detected by flow cytometry for RBITC with the excitation wavelength and emission wavelength at 488 and 575 nm, respectively.

The viability was detected with Calcein-AM and PI detection. HepG2 cells were treated with ALG NGPs and GL-ALG NGPs at 37 °C for 48 h. The medium was discarded and the cells were incubated with Calcein-AM (2.5 μM) and PI (2 μM) for 30 min at 37 °C firstly, and analyzed by using CLSM. HepG2 cells were cultured with ALG NGPs and GL-ALG NGPs for 48 h, and then the phenol red free DMEM was collected for determination of LDH according to the manufacturer′s protocols. The MTT was added to a final concentration of 0.5 mg/ml in DMEM and incubated for an additional 4 h at 37 °C. The culture medium was discarded, and the formazan precipitates were solubilized with DMSO. The absorbance was measured at 490 nm on a multifunctional microplate reader (FlexStation 3, Molecular Devices, USA).

### Tissue distribution of nanogel particles in H22 hepatocarcinoma tumor-bearing mice

Kunming mice are originated from Swiss mice and exhibit minimal variation in growth and reproductive performance, which have been the most widely used mouse type in biological and pharmacological studies in China, especially in immunological research, such as tumor-bearing mice model 15, 16. The tissue distribution of nanogel particles was investigated by RBITC-ALG NGPs or RBITC-GL-ALG NGPs in normal Kunming mice and H22 hepatocarcinoma tumor-bearing mice. After intravenous administered with RBITC-ALG NGPs or RBITC-GL-ALG NGPs, the heart, liver, spleen, lung, kidney, and tumor were removed at different time points (1, 2, 4, 8, 12 and 24 h), and observed by CRI fluorescence imaging system (Maestro 2, CRI, USA).

### The anti-tumor effect of DOX/ GL-ALG NGPs *in vitro*

#### Cell viability, cytotoxicity and enzyme activity assay on DOX/ GL-ALG NGPs

The HepG2 cells were treated with DOX (1, 0.25, 0.0625 μg/ml), DOX/GL-ALG NGPs (NGPs (84, 21, 5.25 μg/ml) containing equal amounts of DOX (1, 0.25, 0.0625 μg/ml), respectively) for 48 h. The viability, cytotoxicity, and caspase activation events were detected within the same sample by Promega ApoTox-Glo™ Triplex Assay kits (Madison, Wisconsin, USA).

### Real-time RT-PCR analysis

HepG2 cells were collected and total RNA of HepG2 cells was isolated using a Sangon UNIQ-10 column Trizol total RNA extraction kit according to the manufacturer's instructions. The real-time RT-PCR primers were shown in table S1. The reactions were setup following FastStart Universal SYBR Green Master (ROX) (Roche, Germany) on an ABI Prism 7500 Real-Time PCR System. The folds increase or decrease were determined relative to a blank control after normalized to a housekeeping gene using the 2^-ΔΔC^_T_ method 22.

### Western blotting

The protein expressions of Bax, Bcl-2, and Bcl-xL were analyzed by western blotting. HepG2 cells were collected by centrifugation and resuspended in extraction buffer of mammalian protein extraction kit (Sangon, China). Cell debris was removed by centrifugation, and the supernatant was collected to detect the protein concentration by BCA method (Pierce, USA). An amount of 40 mg cellular protein was electro-blotted onto a PVDF membrane following separation on a 10 % SDS-polyacrylamide gel electrophoresis. The PVDF membrane was transferred to incubate with blocking solution (5% skim milk) for 2 hours at room temperature, and then incubated overnight with a 1:1000 dilution of anti-Bax, anti-Bcl-2, anti-Bcl-xL, anti-β-actin antibody at 4 °C. Blots were washed five times with Tween 20/Tris-buffered saline (TTBS) and then incubated with a 1:1000 dilution of horseradish peroxidase-conjugated secondary antibody for 1 h at room temperature. Blots were washed five times with TTBS again and then developed by Horseradish peroxidase substrate (Millipore Corporation, USA) and data were captured by exposure to Kodak BioMax Light films.

### Anti-tumor activity of the DOX/ GL-ALG NGPs *in vivo*

The tumor growth inhibitory activities of DOX/ GL-ALG NGPs were assessed in mice bearing H22 liver tumors. The H22 liver tumor models were established according to the technique reported by Shi et al 15. Briefly, Kunming mice with the normal immune system were injected subcutaneously in the right limb armpits with 0.1 ml of a cell suspension containing 2×10^6^ cells. Ten days after the tumor inoculation, the mice were divided into six groups (n = 5), intravenous administered with 0.9% saline (control), ALG NGPs, GL-ALG NGPs, DOX (4 mg/kg), DOX-ALG NGPs (4 mg/kg calculated by free DOX dose) or the DOX/GL-ALG NGPs (4 mg/kg calculated by free DOX dose) in a volume of 0.2 ml. The body weights of the mice were measured daily. The tumor volume of each mouse was monitored, and the tumor volume was calculated as follows: Tumor volume (mm^3^) =width^2^ ×length/2. The antitumor activity was evaluated in terms of tumor weight (g) and volume (mm^3^).

At the time of sacrifice, tumor, liver, and heart were removed and fixed in 10% neutral formalin. The fixed tissue was dehydrated, embedded in paraffin, sectioned, and stained with hematoxylin and eosin (H&E). The stained sections of each test sample were examined using light microscopy (OLYMPUS, Japan). Slides of the tumor were deparaffinized and rehydrated then immunofluorescence-stained using PCNA, Ki67 antibody and Immunofluorescence Detection Kit (Sangon Biotech, Co., Ltd., Shanghai, China) and TUNEL assay kit (Beyotime Biotech, Shanghai, China) according to the manufacturer's instruction.

### Statistical Analysis

All values were represented as mean ±SD excluding results given in pharmacokinetic study in rats that were expressed with mean ± SE. Statistical analysis was performed with Origin Pro 8.5 software by the Student's t-test or one-way ANOVA. The value of *p* < 0.05 or *p* < 0.01 was considered statistically significant.

## Results and Discussion

### Preparation and characterization of DOX/GL-ALG nanogel particles (NGPs)

**Figure 1:**
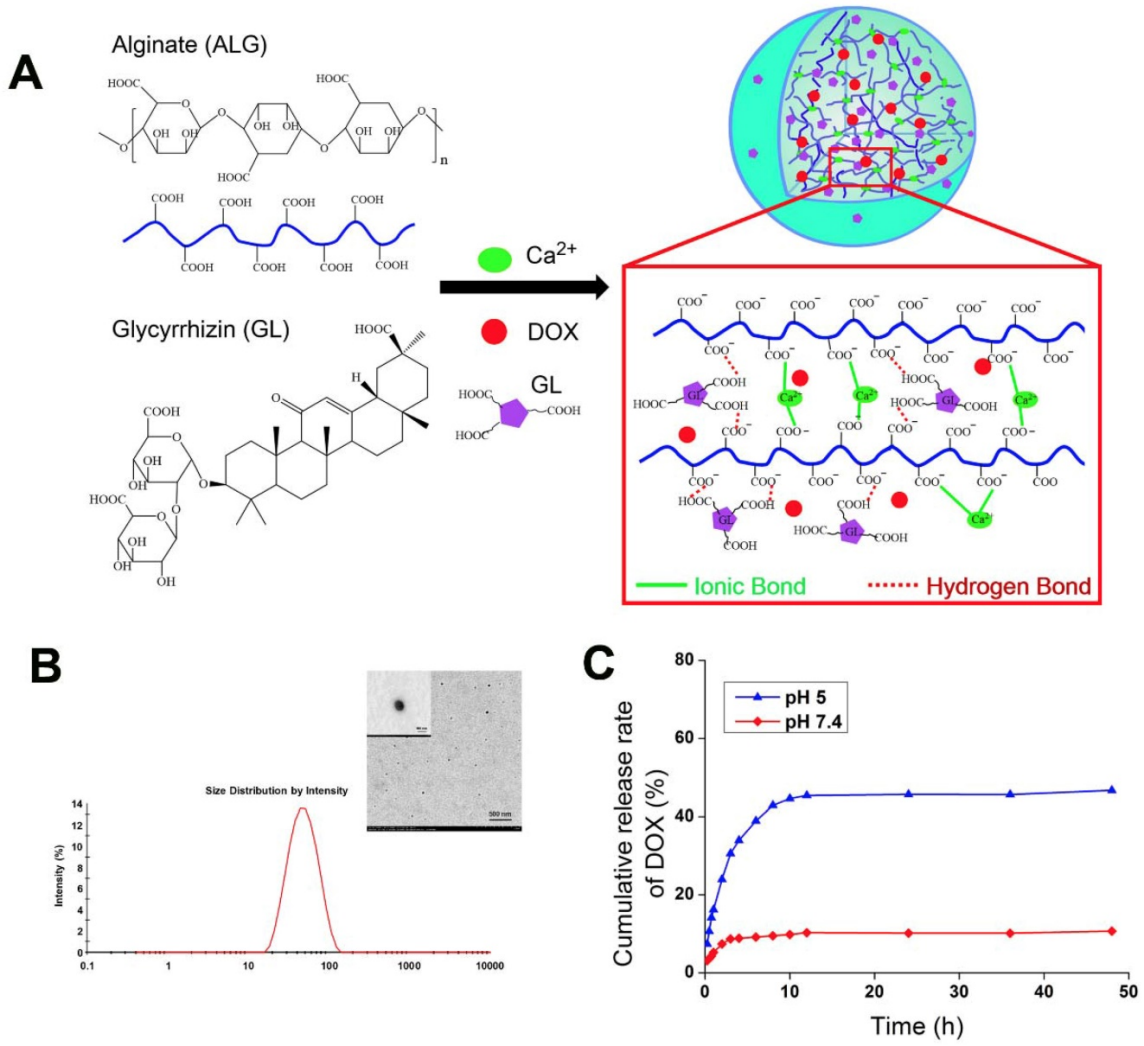
** Preparation and properties of DOX/GL-ALG NGPs.** Formation and structure of DOX/GL-ALG NGPs (0.5% ALG, 0.1% Ca^2+^ and 0.38% GL, w/v) (A). The particle diameter (63.21±0.63 nm) (B) and sustained release of DOX from DOX/GL-ALG NGPs (C).

The NGPs were fabricated by the intermolecular hydrogen bond (between glycyrrhizin and alginate) and ionic bond (between alginate and calcium) (**Figure 1A**), which were verified by the different storage modulus G' values in **Figure S2A**. And the ionic bonding between Ca^2+^ ions and carboxylic acid groups of ALG played a vital role in the nanogel formation than intermolecular hydrogen bonding between GL and ALG. Moreover, the conformational structure of ALG changed as the addition of GL or calcium, which suggested the effect of intermolecular hydrogen bond (between glycyrrhizin and alginate) and ionic bond (between alginate and calcium) (**Figure S2B-D**). These interactions were further identified by the data of FT-IR, XRD, and DSC as shown in **Figure S2E-G**. The FT-IR data showed that-OH characteristic peak (2950, 2875 cm^-1^) in GL shift to 2925 and 2854 cm^-1^ in GL-ALG NGPs (**Figure S2E**), which suggested the interaction of GL and ALG when the NGPs were prepared. The characteristic diffraction peaks of GL disappeared after the formation of GL-ALG NGPs (**Figure S2F**), which also suggested the interaction of GL and ALG. This phenomenon was found in DSC data (**Figure S2G**). The GL-ALG NGPs were spherical and uniformly distributed under TEM, and has a particle size of approximately 63 nm (**Figure 1B**). Zeta potential is a key factor for evaluating the stability of a colloidal dispersion. The negative surface charge of nanoparticles could reduce the plasma protein bioadhesion and has a low rate of non-specific cellular uptake 23. Due to the carboxyl group of ALG, DOX/GL-ALG NGPs had a strongly negative surface charge (-34.0 ±2.1 mV), which remained virtually unchanged after loading drug compared with blank GL-ALG NGPs.

To investigate the feasibility of GL-ALG NGPs as a drug carrier, DOX as a model drug was encapsulated into the GL-ALG NGPs. The drug-loading capacity is about 1.2% and the encapsulation efficiency is 87.45%. As tumor tissues have a more acidic environment than normal physiological conditions, Phosphate-buffered saline (PBS) solution at pH value 5 and 7.4 is chosen to investigate the release profiles of DOX loaded GL-ALG NGPs (**Figure 1C**). The total amount of DOX released from the GL-ALG NGPs was 10.66 % at pH 7.4 and 46.71% at pH 5, respectively, which may be due to the protonation of amino groups at acidic pH. Lower amounts of DOX were released in the blood (pH~7.4) before transport to the target site, and the abundant drug could be released after reaching the lesion site after entering the cells (mimicking microenvironment in endosomes and lysosomes, pH~5-5.5) via receptor-mediated endocytosis. So the GL-ALG NGPs was pH-responsive and could enhance the therapeutic efficacy on the lesion site and reduce the side-effects of the drug on other normal organization.

### The innate immune response of NGPs

**Figure 2:**
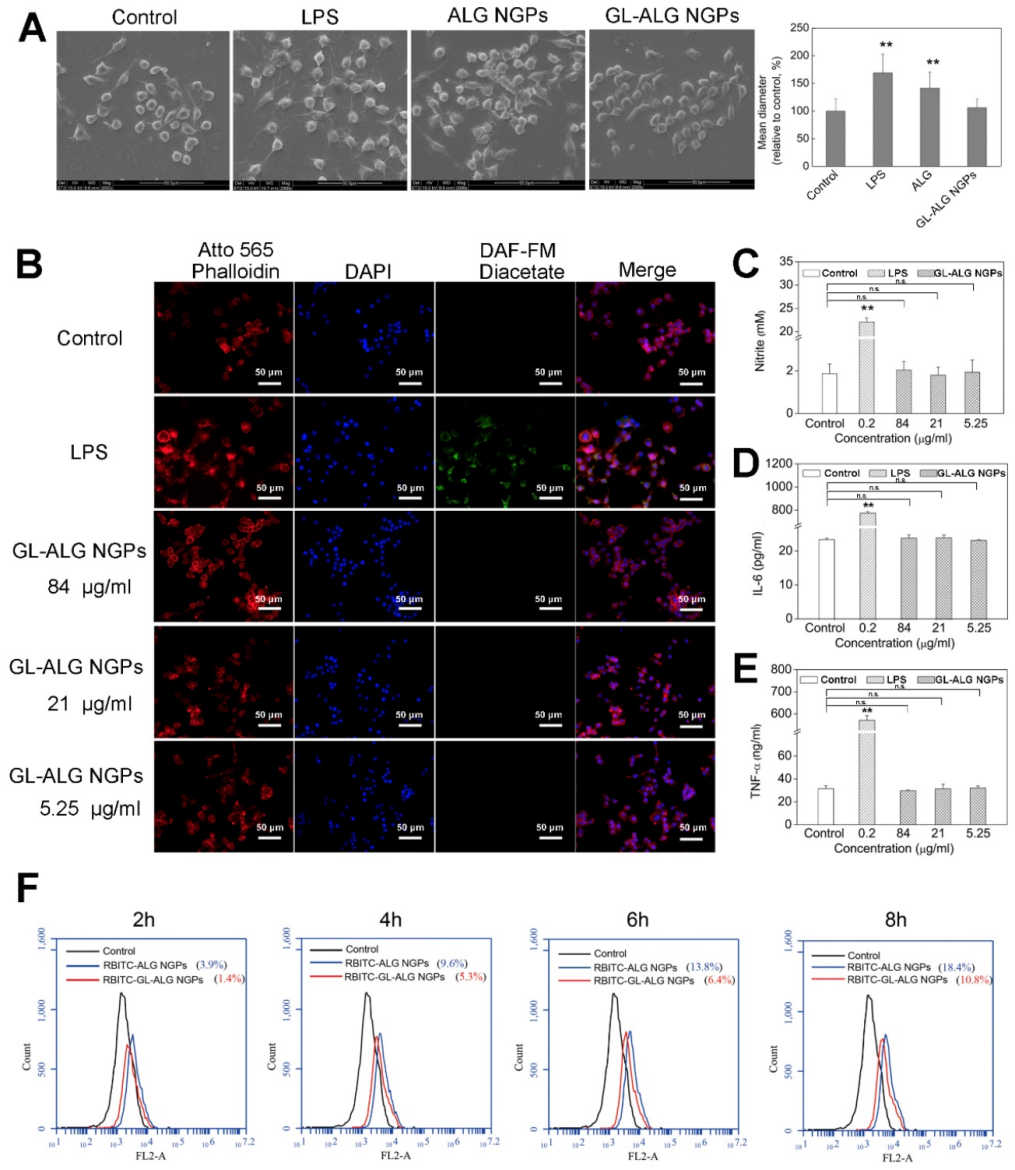
** The immune response and phagocytosis of macrophage (RAW 264.7 cells) on nanogel particles.** Morphologic change of RAW 264.7 cells treatment with ALG and GL-ALG NGPs after 24 h captured by SEM images (A). Intracellular NO production was evaluated with DAF-FM diacetate by inverted fluorescence microscope. Atto 565 Phalloidin (Red fluorescence) was used to dye cytomembrane, and DAPI (Blue fluorescence) was used to dye cell nucleus (B). NO production in culture medium incubated with different concentrations of GL-ALG NGPs, the LPS (0.2 μg/ml) was used as a positive control (C). IL-6 production in culture medium incubated with different concentrations of GL-ALG NGPs (D). TNF-α production in culture medium incubated with different concentrations of GL-ALG NGPs (E). A quantitative determination of cellular phagocytosis of RBITC labeled ALG NGPs and GL-ALG NGPs by flow cytometry on RAW 264.7 cells at 2, 4, 6 and 8 h. The RAW 264.7 cells with the treatment of culture medium were used as the control group (F). ^**^p < 0.01, compared with control group, n.s., not statistically significant.

Macrophages play an important role in host defense through the release of factors such as NO, prostaglandin mediators, and cytokines involved in the immune response, which could recruit more macrophages to remove the foreign substances in the body 24. The RAW 264.7 cells, which are one kind of mouse macrophage cell line, are usually chosen to investigate the response of the nanoparticles by the innate immune system 25. LPS is one of the most powerful activators of macrophages, which was used as the positive control in this study. SEM images depicting cell morphology were obtained in** Figure 2A**. In the control group, the cells were observed with the normal state exhibiting a round-shaped morphology. But cell morphology and mean diameter changed dramatically with LPS stimulation, and many cells exhibited spread morphology, which represented the activated state. The cells treated with GL-ALG NGPs were shown as the almost invariably round shape with fewer cells exhibiting deformed morphology, and the mean diameter of cells almost was similar to the control group. NO was recognized as a mediator and regulator of inflammatory responses with high amounts of production in activated inflammatory cells 26. As shown in **Figure 2B**, the intracellular NO production labeled with DAF-FM diacetate suggested that the strong fluorescence intensity was observed by LPS treatment, and there was almost no fluorescence observed by treating with GL-ALG NGPs. Concentrations of nitrite accumulated in the culture medium were estimated as an index for secretory NO production. LPS could induce the NO production significantly compared to control group, and different concentrations of GL-ALG NGPs have no effect on inducing the NO production (n.s., compared with the control group) (**Figure 2C**). In the immune inflammation progress, a series of cytokines and mediators contributed to evocation and regression of immune response. IL-6 and TNF-α were the critical cytokines involved in immune inflammation progress 27, 28. The LPS treatment significantly induced higher secretion levels of IL-6 and TNF-α than the control group. Different from the positive control (LPS) with high activation, GL-ALG NGPs -treated cells showed little activation up to 84 μg/ml on both IL-6 and TNF-α (n.s., compared with the control group) (**Figure 2D-E**).

Fluorescently labeled nanoparticles can provide a rapid, simple and sensitive means to quantify cell-associated nanoparticles 29. In this study, alginate was labeled with RBITC as a fluorescence probe to observe the cellular phagocytosis between RAW 264.7 cells and nanogel particles. The phagocytosis of RAW 264.7 cells on nanogel particles was quantified via flow cytometry. As shown in **Figure 2F**, the efficiency of cellular uptake was reduced significantly with the introduction of GL in ALG NGPs at different time points. The results suggested the GL-mediated ALG NGPs could reduce the phagocytosis of RAW 264.7 cells* in vitro*, which would play an important role in increasing the long circulation *in vivo*.

### Pharmacokinetic study of DOX loaded in GL-ALG NGPs in rats

Although Nano-based drug delivery systems have produced advancements for the cancer therapy, rapid elimination of nanoparticles from the body suggested a common problem when applying these particles for *in vivo* applications: the absorption of plasma proteins and non-specific uptake by the reticular-endothelial system (RES), like macrophage cells 30. The particles will be removed from the blood circulation system quickly, leading to a dramatic decrease in efficiency of nanoparticle-based diagnostics and therapeutics 31. To reduce the uptake of nanoparticles by the macrophage cells, many attempts have been performed by controlling the physicochemical characteristics of the particles 32, 33. The DOX blood concentration-time profiles showed biphasic curves with a first rapid distribution phase followed by a slow elimination phase (**Figure S3**). The blood concentration of DOX loaded in GL-ALG NGPs was significantly higher than free DOX (*p* < 0.01) at the distribution phase. There was also a significant difference between DOX loaded in GL-ALG NGPs and free DOX in the elimination phase. The main pharmacokinetic parameters calculated using a bi-compartmental pharmacokinetic analysis were summarized in **Table S2**. The distribution half-life (t1/2α) of DOX loaded in GL-ALG NGPs was 7.5-fold higher than that of free DOX (**Table S2**, *p* < 0.01). The half-life (t1/2β) of DOX loaded in GL-ALG NGPs was increased by 4.5 fold in comparison to free DOX.

The increase of blood circulation time on DOX loaded GL-ALG NGPs was also evidenced by the increase of AUC value and the decrease of the total clearance (Cl) in comparison with free DOX (Table S2, *p* <0.01). The AUC_0-24_ and AUC_0-∞_ values of free DOX (1.1 ± 0.4 mg/L*h, 3.4±0.6 mg/L*h) and DOX loaded in GL-ALG NGPs (9.8 ± 0.4 mg/L*h, 44.6 ± 1.6 mg/L*h) further demonstrated the long circulation property of GL-ALG NGPs. The volume of distribution (Vd) of DOX loaded in GL-ALG NGPs was significantly smaller than that of free DOX (*p*<0.01), which showed a less extensive distribution of DOX in tissue when it was administered with DOX loaded in GL-ALG NGPs. In order to investigate the long circulation of nanogel particles with introducing GL, we then compared the change in the kinetics of circulation between DOX/GL-ALG NGPs and DOX-ALG NGPs (**Figure S3**). The distribution half-life (t1/2α) of DOX loaded in GL-ALG NGPs was 5-fold higher than that of DOX loaded in ALG NGPs. The AUC_0-24_ and AUC_0-∞_ values of DOX loaded in GL-ALG NGPs increased about 2.8 and 4.7 folds compared to DOX loaded in ALG NGPs, respectively. These results indicated that ALG NGPs with introducing GL attenuated the rapid uptake by the macrophage phagocytosis of the bloodstream, which prolonged retention period of DOX in the systemic circulation.

### The active targeting effect of GL-ALG NGPs on hepatocellular carcinoma cell (HepG2) and H22 tumor-bearing mice

**Figure 3:**
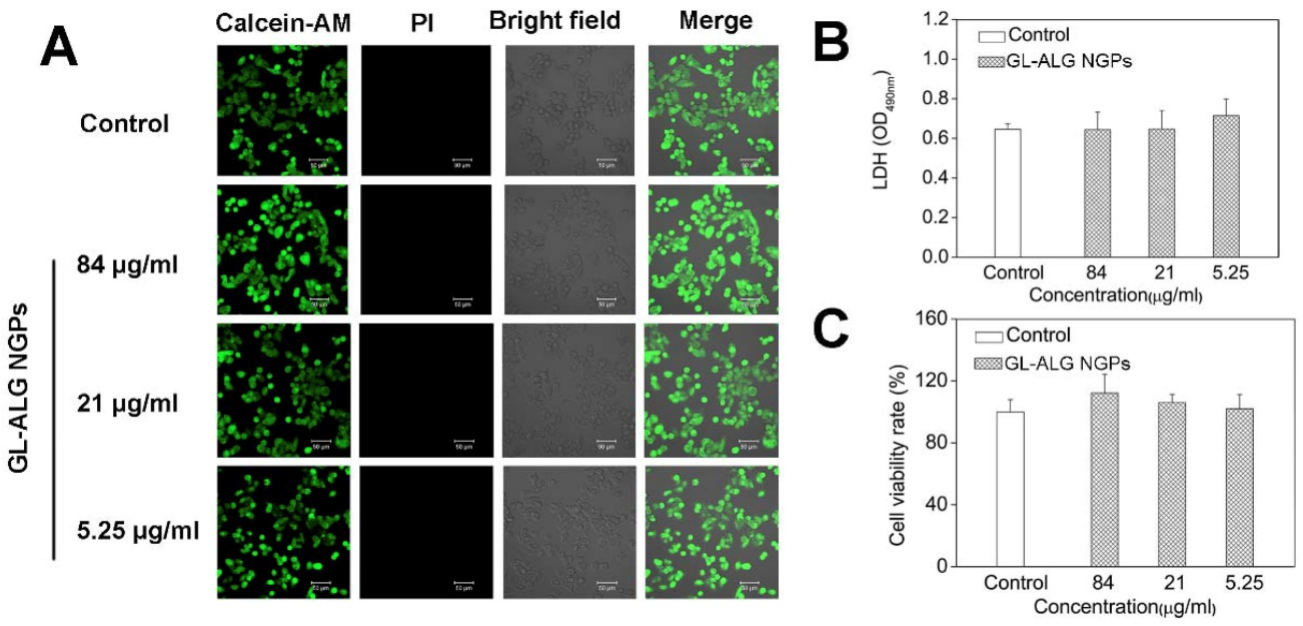
** Cell viability of GL-ALG NGPs on hepatocellular carcinoma cell (HepG2).** The viability of HepG2 cells cultured with GL-ALG NGPs was detected by an inverted fluorescence microscope. Calcein AM (Green fluorescence) and PI (Red fluorescence) were used to dye intact live and dead cells, respectively (A). LDH secretion was detected with LDH assay kit (B), Cell viability was detected by MTT assay (C).

Before investigating the cellular uptake of HepG2 cells on GL-ALG NGPs, we first detected the cell viability of GL-ALG NGPs on HepG2 cells with fluorescence imaging, MTT, and LDH assay. The fluorescence assay using calcein-AM and PI showed that the different concentration of GL-ALG NGPs (84, 21, 5.25 μg/ml) did not affect the cell viability compared to control group (**Figure 3A**). The secretion of LDH was an indicator of cytotoxicity since the integrity of the cell membrane was destroyed in the apoptosis or necrosis of cell. The production of LDH was not increased in GL-ALG NGPs groups compared to the control group (**Figure 3B**). The cell viability of HepG2 cells was also confirmed by MTT assay (**Figure 3C**). The results suggested that the GL-ALG NGPs were safe for liver drug delivery.

**Figure 4:**
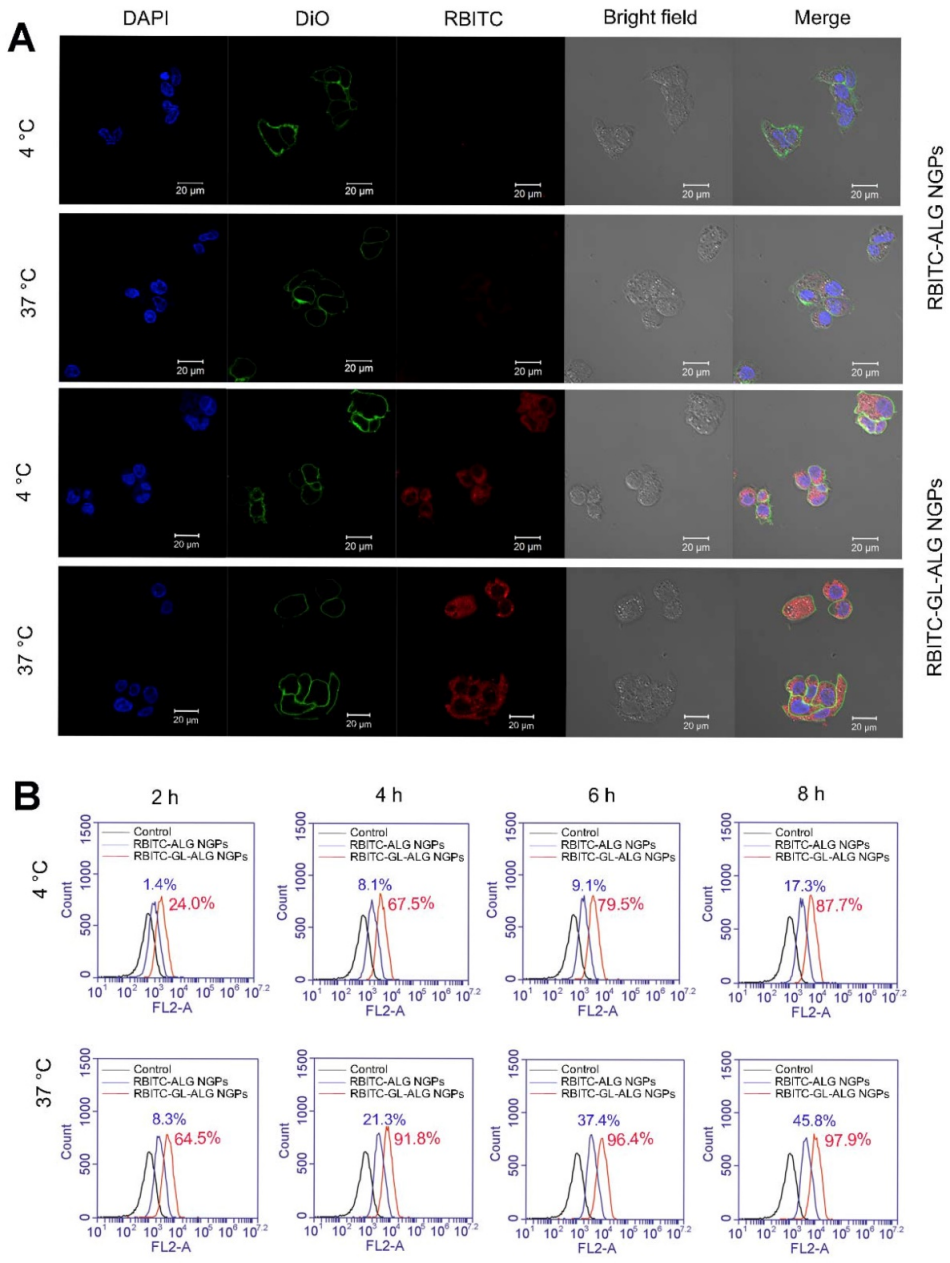
** Glycyrrhizin-mediated active targeting effect of ALG NGPs on HepG2.** Cellular uptake of HepG2 cells on ALG NGPs and GL-ALG NGPs at 4 or 37 °C analyzed by laser scanning confocal microscope. GL-ALG NGPs was labeled with rhodamin B isothiocyanate (RBITC) (Red fluorescence). DiO (green fluorescence) was used to dye cytomembrane, and DAPI (Blue fluorescence) was used to stain the cell nucleus (A). A quantitative determination of cellular phagocytosis of RBITC labeled ALG NGPs and GL-ALG NGPs by flow cytometry at 4 and 37 °C on HepG2 cells at 2, 4, 6 and 8 h. The HepG2 cells without treatment were used as the control group (B).

In order to investigate whether the uptake of nanogel particles into the cells was receptor-mediated dependent, we incubated the nanogel particles with HepG2 cells at 4 °C or 37 °C. After incubating the HepG2 cells with RBITC-ALG NGPs at 4 °C for 4 h, there was little intracellular fluorescence observed in HepG2 cells. The endocytosis was inhibited at 4 °C as the cellular uptake of nanogel particles was energy-dependent, however, strong intracellular fluorescence was observed in HepG2 cells treating with RBITC-GL-ALG NGPs at 4 °C (**Figure 4A**). This was due to the abundant receptors on hepatocytes membranes for GL, which improved the cellular uptake of nanogel particles by active targeting effect without energy-dependent. Moreover, when varying the culture condition at 37 °C, the weak intracellular fluorescence could be observed in HepG2 cells treatment with RBITC-ALG NGPs, suggesting that the passive endocytosis of RBITC-ALG NGPs was rare. The tendency was changed with the introduction of GL into ALG NGPs, stronger intracellular fluorescence was observed in HepG2 cells compared to treatment of RBITC-ALG NGPs. This intensive cellular uptake was the combination of passive endocytosis and active targeting effect, and active targeting effect played a leading role in phagocytosis. The phagocytosis of HepG2 cells on nanogel particles was further quantified via flow cytometry. As shown in **Figure 4B**, the efficiency of cellular uptake was enhanced about 8.3 times with the introduction of GL in ALG NGPs. As time goes on, there was 87.7% of RBITC-GL-ALG NGPs and 17.3% of RBITC-ALG NGPs observed in HepG2 cells after 8 h. Moreover, we investigated the normal cell culture condition at 37 °C, in which the passive endocytosis and GL-mediated receptor targeting effect worked together on the uptake of nanogel particles. The efficiency of cellular uptake was still enhanced about 4.3 times on RBITC-GL-ALG NGPs compared to RBITC-ALG NGPs after 4 h. The results suggested the GL-mediated active targeting played a leading role in cellular uptake of nanogel particles *in vitro*.

**Figure 5:**
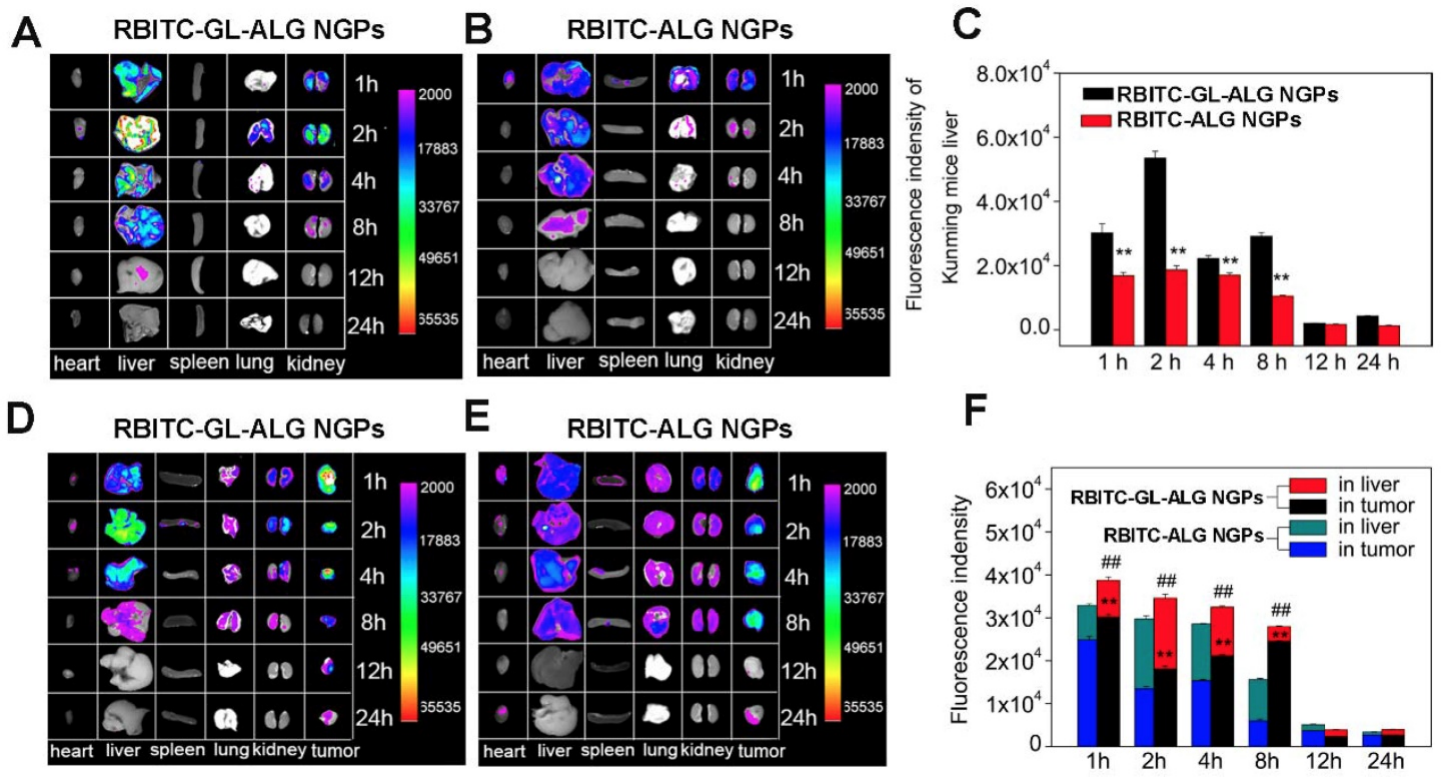
** Tissue distribution of RBITC labeled ALG NGPs and GL-ALG NGPs in normal mice and H22 hepatocarcinoma tumor-bearing mice.** After intravenous administered with RBITC-labelled ALG (A) and GL-ALG NGPs (B) (4 mg per mice), the heart, liver, spleen, lung, and kidney in normal mice were observed at different time point. Relative quantitative analysis of RBITC-labelled ALG NGPs and GL-ALG NGPs accumulation in the liver (C). ^**^p < 0.01, compared with RBITC-labelled ALG NGPs in liver. After intravenous administered with RBITC-labelled ALG (D) and GL-ALG NGPs (E), the heart, liver, spleen, lung, kidney, and tumor in H22 hepatocarcinoma tumor-bearing mice were observed at different time point. Relative quantitative analysis of RBITC-labelled ALG NGPs and GL-ALG NGPs accumulation in liver and tumor (F). ^**^p < 0.01, compared with RBITC-labelled ALG NGPs in tumor, ^##^*p*< 0.01, compared with RBITC-labelled ALG NGPs in liver and tumor.

To evaluate nanoparticle efficacy, an animal model must be chosen which has an immune status similar to the actual disease 34. Firstly, we investigated the active targeting effect of nanogel particles in Kunming mice with the normal immune system. The fluorescence intensity of nanogel particles in different tissue was shown in **Figure 5A**. After 1 h of injecting RBITC-ALG NGPs or RBITC-GL-ALG NGPs, the strongest fluorescence intensity was found in the liver and kidney. The fluorescence intensity of GL-ALG NGPs was twice as much as ALG NGPs in the liver even after 8 h, which might be the active targeting effect of GL in ALG NGPs resulting in more accumulation than ALG NGPs in the liver (**Figure 5C**). Secondly, to investigate whether there was the targeting effect of NGPs in the tumor, RBITC-ALG or RBITC-GL-ALG NGPs were injected into the tail vein of H22 tumor-bearing mice, which could be more close to the clinical symptom rather than BALB/c nude mice with immune deficiency. The result of fluorescence analysis showed that more GL-ALG NGPs assembled in tumor than ALG NGPs (**Figure 5F**). When the RBITC-ALG or RBITC-GL-ALG NGPs were injected in H22 tumor-bearing mice, more RBITC-GL-ALG NGPs accumulated in tumor than RBITC-ALG NGPs in H22 tumor-bearing mice, which led to less RBITC-GL-ALG NGPs accumulation in the liver. So this is the reason why it does not seem like there is a difference in the liver accumulation for RBITC-GL-ALG NGPs vs RBITC-ALG NGPs for times <8h. Especially after 8 h of injection, the accumulation efficiency of GL-ALG NGPs in the tumor was almost 4.08 times than ALG NGPs. Furthermore, the total fluorescence intensity was increased significantly by GL-ALG NGPs injection compared to ALG NGPs injection at different points in liver and tumor (*p*<0.01, compared to ALG NGPs), and the other tissue showed weaker fluorescence intensity. The surface of the liver and hepatoma cell exists lots of GL receptor 14, 35, which was introduced for GL-mediated liver and hepatoma cell-targeted drug delivery systems 36. When RBITC-GL-ALG NGPs were injected, GL-mediated active targeting effect (liver and hepatoma cell targeting effect) resulted in more nanogels to the liver or tumor site. Thus, AUC for RBITC-GL-ALG NGPs was greater than that for RBITC-ALG nanoparticles. Similarly, the total fluorescence intensity was increased significantly by GL-ALG NGPs injection compared to ALG NGPs injection at different points in the liver and tumor. The fluorescence intensity decreased as the metabolism of the kidney, and little fluorescence intensity was observed after 24 h of NGPs injection. These indicated that the prepared GL-ALG NGPs exhibited good liver targeting ability due to both passive targeting via the enhanced permeability and retention effects, and the active targeting ability of GL.

### The anti-tumor effects of DOX/ GL-ALG NGPs *in vitro*

The cytotoxicity was detected by ApoTox-Glo™ Triplex Assay (Promega, USA) which combined three assay chemistries to assess viability, cytotoxicity, and caspase activation events within a single assay well. DOX (1, 0.25, 0.0625 μg/ml) and DOX/GL-ALG NGPs (NGPs (84, 21, 5.25 μg/ml) containing equal amounts of DOX (1, 0.25, 0.0625 μg/ml), respectively) could not only decrease the viability, but increase the toxicity of hepatoma cells significantly than control group (*p*<0.01). Moreover, compared to free DOX, the DOX/GL-ALG NGPs decreased the viability and increase the toxicity of hepatoma cells significantly (*p*<0.05 or *p*<0.01) (**Figure S4**). It was interesting that DOX/GL-ALG NGPs showed increased cytostatic activity in comparison to free DOX since less than 10.67% of DOX was released from the NGPs at pH 7.4 within 48 h. The involvement of P-glycoprotein might restrict the transport of the free DOX, which could be pumped out from the cells as the P-glycoprotein substrates 37. The previous study suggested that DOX-containing in anionic liposomal nanoparticles could inhibit P-glycoprotein activity as a vehicle particularly indicated against drug-resistant tumors 38. As the receptor-mediated drug targeting effect of GL in ALG NGPs, the GL-ALG NGPs could aggregate into the endosomes to release drugs reducing the efflux effect of P-glycoprotein, which enhanced the drug efficacy of the cytotoxicity for hepatoma cells.

**Figure 6:**
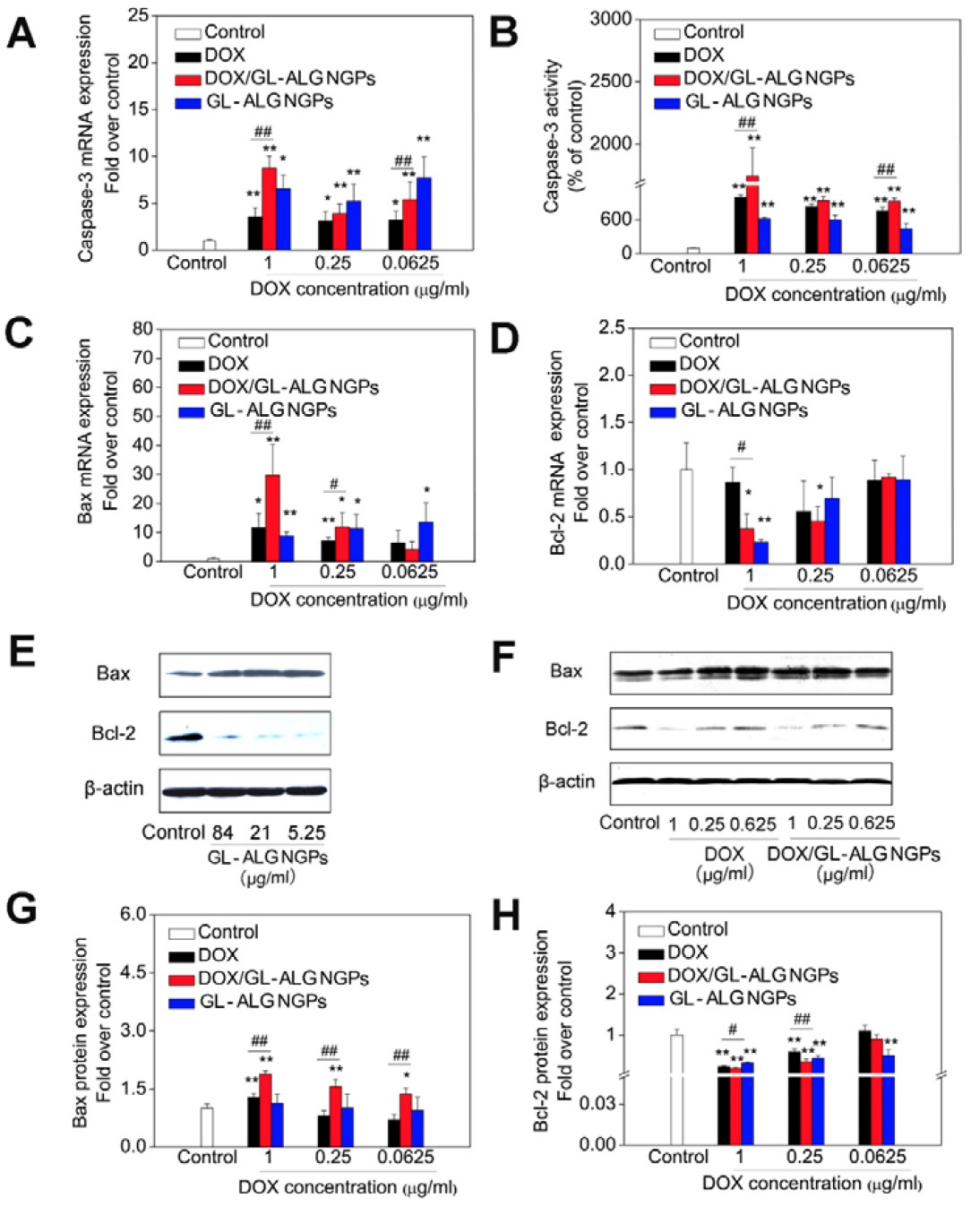
** The antitumor effects of DOX/GL-ALG NGPs *in vitro***. The HepG2 cells were treated with GL-ALG NGPs and DOX/GL-ALG NGPs for 48 h. The mRNA expression of caspase-3 and the activity of caspase-3 were shown in (A) and (B), separately. The mRNA expression of Bax (C) and Bcl-2 (D) was detected by real-time RT-PCR. The protein expression of Bax, Bcl-2 incubated with GL-ALG NGPs (E) and DOX/GL-ALG NGPs was analyzed by Western Blot. The western blot film was scanned and the intensity (Bax [G], Bcl-2 [H]) was quantified by Image J version 1.51n and normalized to the corresponding β-actin intensity and the controls. ^**^p < 0.01, compared with control group, ^##^*p* < 0.01, compared with free DOX group.

The caspase family plays an important role in the regulation of apoptosis. Activation of caspase is a symbol of apoptosis induction in response to death-inducing signals originating from mitochondria or endoplasmic reticulum 39. As an effector caspase, the level of caspase-3 activation plays an important role in the death-receptor and mitochondrial pathways, which is used extensively in response to the extracellular and intracellular stimulus to induce apoptosis 40. The mRNA expression of caspase-3 was up-regulated significantly by GL-ALG NGPs, DOX and DOX/GL-ALG NGPs compared to control group (*p*<0.05 or *p*<0.01, **Figure 6A**). The GL-ALG NGPs without DOX, DOX and DOX/GL-ALG NGPs had higher efficiency on active the caspase-3 than the control group (*p*<0.01, **Figure 6B**). Although less than 10.67% of free DOX was released from the NGPs within 48 h, the activity of caspase-3 was increased 1.77 times by DOX/GL-ALG NGPs than DOX group.

As one of the most important anti-apoptotic members in the Bcl-2 family, Bcl-2 binds to the pro-apoptotic proteins Bax (Bcl-2-associated X protein) to protect cells against apoptosis by maintaining the integrity of the mitochondrial membrane 41. When Bax is released from their anti-apoptotic counterparts, they can cause cell death by activation of caspase-3 and-9 42. Thus, the balance of bcl-2 family members is crucial to the apoptotic trigger, and the anti-apoptotic proteins of the Bcl-2 family are validated drug targets for cancer treatment 43. As shown in **Figure 6 C-D**, the mRNA expression of Bax was upregulated significantly by the GL-ALG NGPs, DOX, and DOX/GL-ALG NGPs, whereas Bcl-2 was downregulated significantly compared to control group. This had the effect of greatly increasing the ratio of the proapoptotic Bax to the antiapoptotic Bcl-2 messenger (*p*<0.01, **Figure S5**). Western blotting analysis (**Figure 6E-F**) of cells treated with DOX, DOX/GL-ALG NGPs indicated that the protein expression of Bax increased significantly compared to control group in a concentration-dependent manner (*p*<0.05 or *p*<0.01). There was also a significant difference between the GL-ALG NGPs and the control group (*p*<0.01). In contrast, considerable decrease in Bcl-2 protein levels was observed, which resulted in a significant increase in the Bax/Bcl-2 ratio in GL-ALG NGPs, DOX and DOX/GL-ALG NGPs-treated HepG2 cells compared with control group (1.82-fold higher in DOX/GL-ALG NGPs at concentration of 1 μg/ml than that of free DOX, *p*<0.01). This was consistent with the corresponding change observed for mRNA expression. Furthermore, the mRNA and protein expression of Bcl-xL was also investigated. The results showed that the mRNA expression of Bcl-xL was downregulated significantly, however, the protein expression was not downregulated by the GL-ALG NGPs, DOX and DOX/GL-ALG NGPs significantly in comparison with the control group (**Figure S6**). Reports indicated that the augment of the Bax/Bcl-2 ratio might contribute to apoptosis via caspases activation in the mitochondria pathway 44. This study revealed that Bcl-2 expression was significantly inhibited while Bax expression was relatively constant, suggesting that the DOX/GL-ALG NGPs induced apoptosis by shifting the Bax/Bcl-2 ratio in favor of apoptosis.

### The anti-tumor effect of DOX/ GL-ALG NGPs* in vivo*

**Figure 7:**
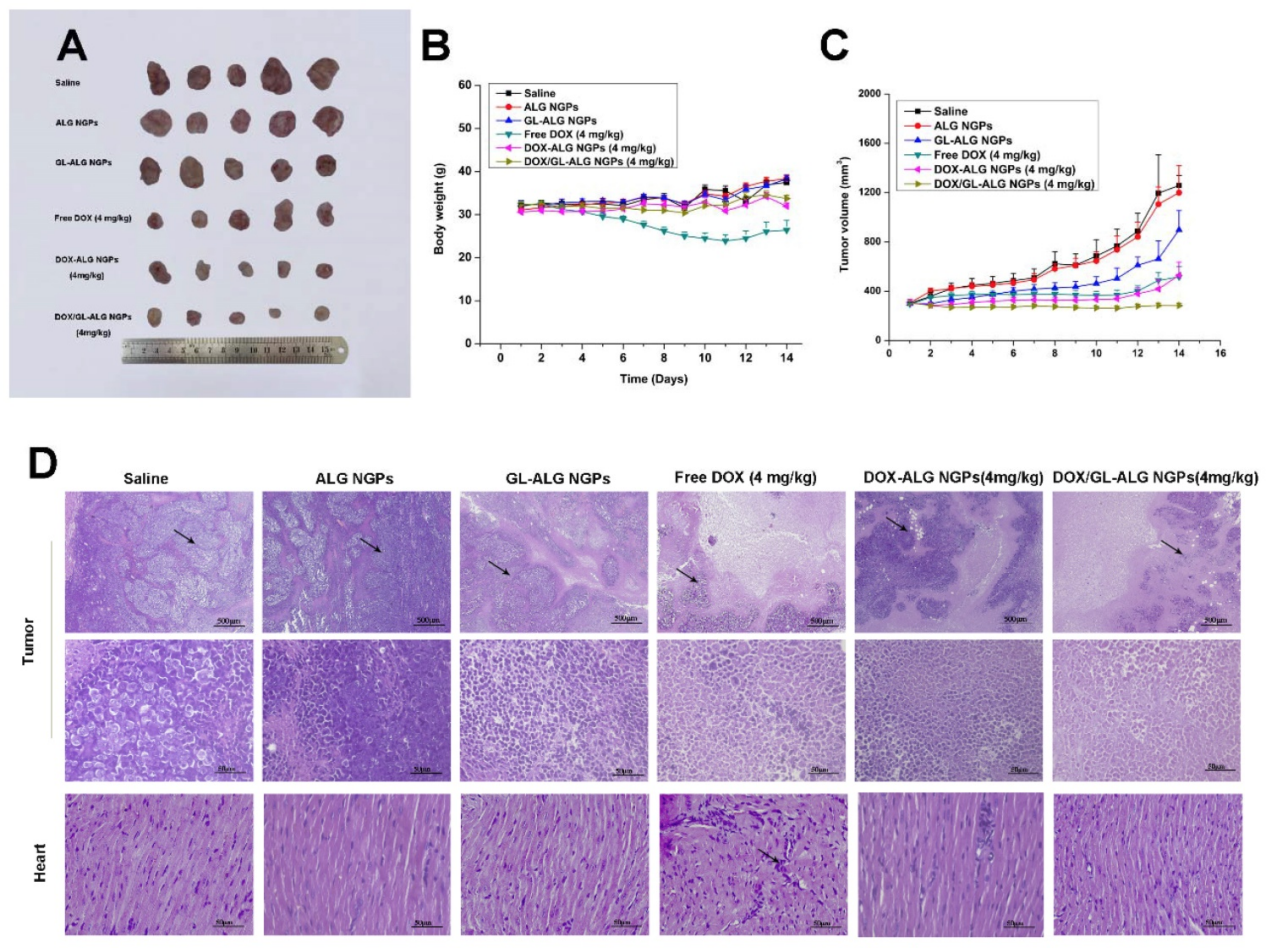
** The antitumor effects of DOX/GL-ALG NGPs in H22 hepatocarcinoma tumor-bearing mice.** Representative Tumors separated from animals received an intravenous injection of saline, ALG NGPs, GL-ALG NGPs, DOX (4 mg/kg), DOX -ALG NGPs (4 mg/kg), DOX/GL-ALG NGPs (4 mg/kg) (A). Effect of DOX/GL-ALG NGPs on body weight of mice (n=5) (B). Effect of DOX/GL-ALG NGPs on tumor volume (n=5) (C). Sections of the tumor and myocardial tissues taken from animal received an intravenous injection of saline, ALG NGPs, GL-ALG NGPs, DOX (4 mg/kg), DOX-ALG NGPs (4 mg/kg), DOX/GL-ALG NGPs (4 mg/kg) (D).

The *in vivo* antitumor activities of ALG NGPs, GL-ALG NGPs, DOX (a dose of 4 mg DOX/kg body weight), DOX-ALG NGPs (dose of 4 mg DOX loaded in ALG NGPs/kg body weight) and DOX/GL-ALG NGPs (dose of 4 mg DOX loaded in GL-ALG NGPs/kg body weight) were evaluated in Kunming mice bearing H22 liver tumors (**Figure 7**). Representative photographs of the excised H22 liver tumors provided an additional, direct visual representation of the tumor-suppression effect (**Figure 7A**). The tumor growth inhibition (based on the tumor volumes) was significant after treatment with DOX (4 mg/kg), DOX-ALG NGPs (4 mg/kg) and DOX/ GL-ALG NGPs (4 mg/kg) in 14 days, respectively (**Figure 7C**). It was worth noting that GL-ALG NGPs also could inhibit the tumor growth significantly compared to the treatment of normal saline (*p*<0.01). Among the three drug administration groups, the DOX/GL-ALG NGPs exhibited greater antitumor effects than free DOX or DOX-ALG NGPs. Multidrug resistance is often associated with an ATP-dependent decrease in cellular drug accumulation with the overexpression of certain ATP-binding cassette transporter proteins (ABC) in tumor cells 45. The multidrug resistance protein 1 (MRP1), one of ABC proteins, is expressed in tissues and important for absorption (e.g., lung and gut) and metabolism and elimination (liver and kidney). Furthermore, the previous study revealed that the mRNA expression of MRP1 was increased in hepatocellular carcinoma compared with the surrounding tissue 46. DOX is the substrate of MRP1 47, and glycyrrhizin is the inhibitor of MRP-1 48. Therefore, The GL in ALG NGPs could inhibit the activity of MRP1, and more DOX could be delivered to tumor tissue.

**Figure 8:**
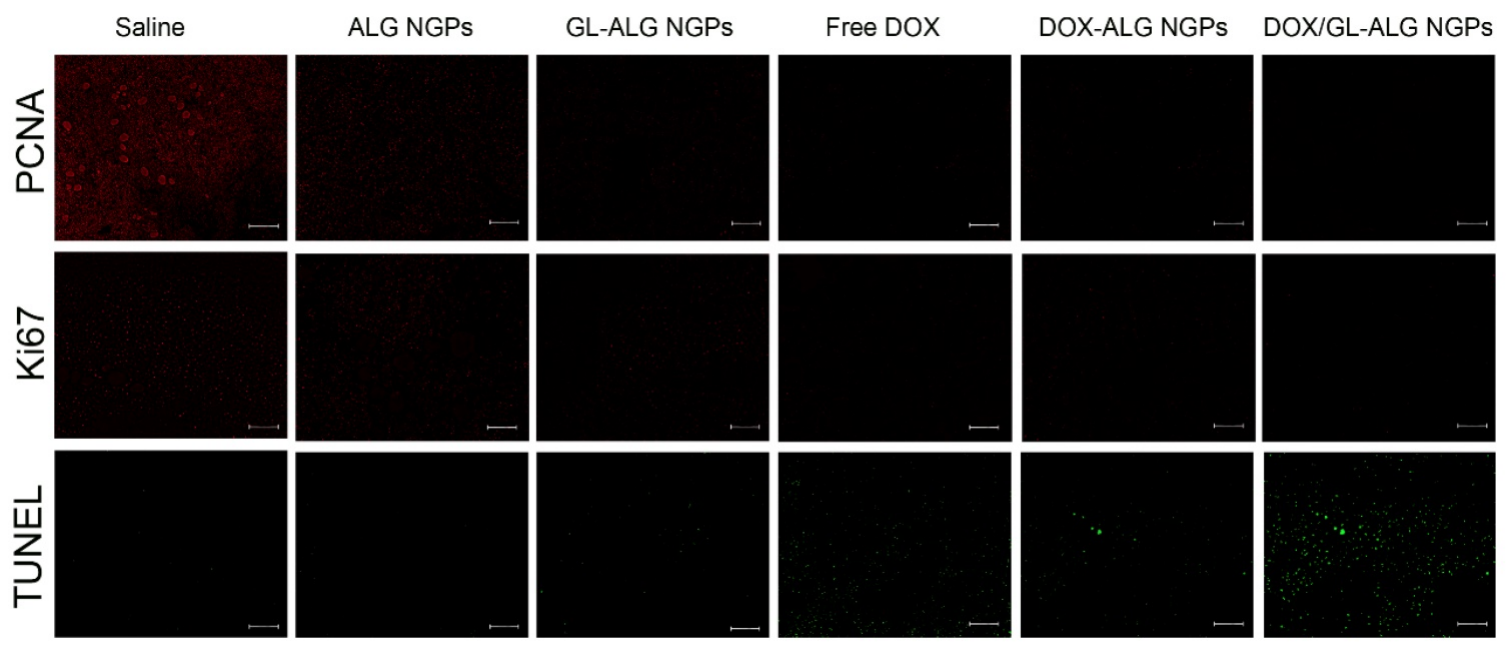
** PCNA staining, Ki67 staining and TUNEL staining of tumor sections after different treatments (saline, ALG NGPs, GL-ALG NGPs, Free DOX (4 mg/kg), DOX-ALG NGPs (4 mg/kg), DOX/GL-ALG NGPs (4 mg/kg)).** (Scale bar: 100 μm)

The change in body weight of the mice is an important index for evaluating the systemic toxicity 49. The variation in body weight of the mice was shown in **Figure 7B**. The mice treated with DOX displayed obvious body weight reduction, suggesting its toxic effect on animals. The body weight of mice in GL-ALG NGPs groups did not differ greatly from that of saline, indicating that the GL-ALG NGPs did not exhibit severe systemic toxicity. In contrast, the body weight loss was reversed by the DOX/GL-ALG NGPs (4 mg/kg) compared to free DOX (4 mg/kg) groups. This suggested the GL-ALG NGPs could reduce the systemic toxicity for long-term application. Histological staining of morphological changes in tumors was shown in **Figure 7D**. In the normal saline group, tumor cells were histiocytoid in shape and arranged tightly. The nucleus of tumor cells was large and apparent with the common characteristics of H22 cells, indicating the successful establishment of H22 liver tumor model. However, in GL-ALG NGPs group, DOX group, DOX-ALG NGPs and DOX/GL-ALG NGPs group, the proportion of tumor area was reduced. The tumor cells were arranged loosely and surrounded by normal cells with lightly stained or condensed nuclei, and this indicated that the growth of H22 liver tumor was inhibited. We next investigated the tumor cell growth activity using the proliferating cell nuclear antigen (PCNA) staining and Ki67 staining after 14 days of treatment (**Figure 8A-B**). The high fluorescence intensity of PCNA and Ki67 positive tumor cells indicated the high proliferation ability. Compared with the saline treatment, free DOX, DOX-ALG NGPs, and DOX/GL-ALG NGPs exhibited lower positive tumor cells with weaker fluorescence intensity. On the other hand, TUNEL was applied to examine the apoptotic status of tumor cells (**Figure 8C**). The injection of free DOX, DOX-ALG NGPs, and DOX/GL-ALG NGPs remarkably increased TUNEL-positive tumor cells compared with saline treatment, and the DOX/GL-ALG NGPs exhibited the strongest fluorescence intensity than free DOX or DOX-ALG NGPs. DOX/GL-ALG NGPs could inhibit the growth activity of tumor cells and promote apoptotic of tumor cells to enhance antitumor effects.

The therapeutic potential of DOX has been restricted by its toxic side effect, mainly its cardiotoxicity, which results in a narrow therapeutic index due to its high toxicity to healthy tissues 50. Reduction of the drug side effects is still one of the major concerns for developing novel drug delivery carrier systems. Hearts in the normal saline group exhibited normal myocardium architecture and cell distribution. Similarly, in GL-ALG NGPs group, no histopathological feature was observed in the heart tissue. With the application of free DOX, myofibrillar loss and inflammatory cell infiltration (black arrows) were observed, indicated the obvious cardiotoxicity 51. In contrast to the DOX group, there was little histopathological feature observed in DOX-ALG NGPs, and no obvious histopathological abnormalities were observed following the treatment of DOX/GL-ALG NGPs (4 mg/kg), which indicated that the DOX/GL-ALG NGPs delivery system avoided the cardiotoxicity induced by free DOX. The report showed that glycyrrhizin could alleviate rat myocardial I/R-induced injury via blocking the phospho-JNK/Bax pathway 12. One reason was that the DOX/GL-ALG NGPs could perform targeting delivery of DOX to the tumor, which decreased the accumulation of DOX in heart to avoid the cardiotoxicity. The other reason might be the glycyrrhizin released from the nanogel particles performing the myocardial protection, which was proven by combined administration of soluble GL along with DOX compared to free DOX. GL could reduce the cardiotoxicity induced by free DOX (**Figure S7**). Furthermore, the hepatotoxicity of nanogel particles was also investigated. As shown in **Figure S8**, free DOX treatment resulted in hepatic damage including macro- and microvesicular steatosis and bile stasis. The histopathological feature was not observed in other groups. In further to investigate the hepatotoxicity, the liver enzyme levels of ALT and AST were also detected. The levels of ALT and AST were increased significantly by free DOX treatment compared to saline treatment, and DOX/GL-ALG NGPs could decrease the hepatotoxicity induced by DOX. These results suggested DOX/GL-ALG NGPs were safe for targeting treatment of hepatocellular carcinoma.

## Conclusion

NDDS for cancer therapy was considered as an intruder by the innate immune system and could be readily recognized and engulfed by macrophage cells. In this study, glycyrrhizin mediated nanogel particles could avoid activating the innate immune system and phagocytosis of macrophage to prolong the blood circulation system. GL was not only as one drug loaded in ALG NGPs, but could participate in the construction of NGPs by an intermolecular hydrogen bond between GL and ALG. Furthermore, the bioavailability of DOX was improved significantly by GL-ALG NGPs than free DOX. The mice with normal immune functions used in constructing the tumor-bearing mice instead of the nude mice with immunodeficiency also indicated the good biocompatibility of NGPs. The combination therapy of DOX and GL in ALG NGPs not only increase the active targeting effect of tumor therapy but exert significant anti-tumor effect via regulating apoptosis pathway, moreover, decrease the cardiotoxicity of free DOX as the cardioprotective effect of GL. These results would provide a new strategy with the combination therapy of natural products and chemotherapeutic drug for cancer, especially liver cancer.

## Supplementary Material

The real-time RT-PCR oligonucleotide primers (Table S1), pharmacokinetic parameters of DOX after intravenous injection (i.v.) administration to rats in free DOX (f-Dox) and DOX/GL-ALG NGPs (Table S2), the structure of glycyrrhizin (A) and glycyrrhetinic acid (B) (**Figure S1**), the characterization of DOX/GL-ALG NGPs (**Figure S2**), pharmacokinetic profiles in rats after i.v. administration of DOX solution and DOX- DOX/GL-ALG NGPs (**Figure S3**), cell viability and toxicity of DOX/GL-ALG NGPs on hepatocellular carcinoma cells (**Figure S4**), the mRNA and protein expression ratio of Bax and Bcl-2 *in vitro* (**Figure S5**), the effect of DOX/GL-ALG NGPs on the mRNA and protein expression of Bcl-xL (**Figure S6**), the myocardial protection of GL on DOX-induced cardiotoxicity in mice (**Figure S7**), the effect of DOX/GL-ALG NGPs on hepatotoxicity, AST and ALT levels in mice (**Figure S8**), cell viability of DOX/GL-ALG NGPs on RAW264.7 cells and L929 cells (**Figure S9**).

## Abbreviations

NDDS: Nanocarrier drug delivery systems
NGPs: nanogel particles
HCC: Hepatocellular carcinoma
RES: reticular-endothelial system
HepG2: Human hepatocellular carcinoma
PIT: phase inversion temperature
DSC: Differential scanning calorimetry
ABC: ATP-binding cassette transporter proteins
MRP1: multidrug resistance protein 1
