# Supplementary material for: Co-delivery of glycyrrhizin and doxorubicin by alginate nanogel particles attenuates the activation of macrophage and enhances the therapeutic efficacy for hepatocellular carcinoma
Source: Theranostics. 2019 Aug 14;9(21):6239–55. doi: 10.7150/thno.35972 (PMC6735516; doi:10.7150/thno.35972)
Supplement: Supplementary file 1 — The real-time RT-PCR oligonucleotide primers (Table S1), pharmacokinetic parameters of DOX after intravenous injection (i.v.) administration to rats in free DOX (f-Dox) and DOX/GL-ALG NGPs (Table S2), the structure of glycyrrhizin (A) and glycyrrhetinic acid (B) (Figure S1), the characterization of DOX/GL-ALG NGPs (Figure S2), pharmacokinetic profiles in rats after i.v. administration of DOX solution and DOX- DOX/GL-ALG NGPs (Figure S3), cell viability and toxicity of DOX/GL-ALG NGPs on hepatocellular carcinoma cells (Figure S4), the mRNA and protein expression ratio of Bax and Bcl-2 in vitro (Figure S5), the effect of DOX/GL-ALG NGPs on the mRNA and protein expression of Bcl-xL (Figure S6), the myocardial protection of GL on DOX-induced cardiotoxicity in mice (Figure S7), the effect of DOX/GL-ALG NGPs on hepatotoxicity, AST and ALT levels in mice (Figure S8), cell viability of DOX/GL-ALG NGPs on RAW264.7 cells and L929 cells (Figure S9). [file thnov09p6239s1.pdf]

Table S1. The real-time RT-PCR oligonucleotide primers.

Gene	Primer	Sequence(5’-3’)	PCR product (bp)
β-actin	Forward	TGTTACCAACTGGGACGACA	165
(NM_007393.3)	Reverse	GGGGTGTTGAAGGTCTCAAA	
Bax	Forward	CTGCAGAGGATGATTGCTGA	174
(NM_007527.3)	Reverse	GATCAGCTCGGGCACTTTAG	
Bcl-2	Forward	GGACTTGAAGTGCCATTGGT	127
(NM_177410.2)	Reverse	AGCCCCTCTGTGACAGCTTA	
Caspase-3	Forward	ATGGGAGCAAGTCAGTGGAC	137
(NM_009810.2)	Reverse	CGTACCAGAGCGAGATGACA	

Table S2. Pharmacokinetic parameters of DOX after intravenous injection (i.v.) administration to rats in free DOX (f-Dox) and DOX/GL-ALG NGPs (n = 4).

Parameters	f-DOX	DOX/GL-ALG NGPs
T1/2α (h)	0.06±0.01	0.5±0.0**
T1/2β (h)	3.6±0.1	16.5±0.2**
$AUC_{(0\text{-}24)}$(mg/L*h)	1.1±0.4	9.6±0.4**
$AUC_{(0\text{-}\infty)}$(mg/L*h)	3.4±0.6	44.6±1.6**
Cl (L/h)	5.1±0.5	0.5±0.04**
Vd (L)	8.9±2.3	4.0±0.5**

T1/2α: The distribution half-life

T1/2β: The Elimination half-life.

AUC: Area under the plasma DOX concentration–time curves

Cl: the total clearance

Vd: the volume of distribution

** P<0.01, compared to free DOX.

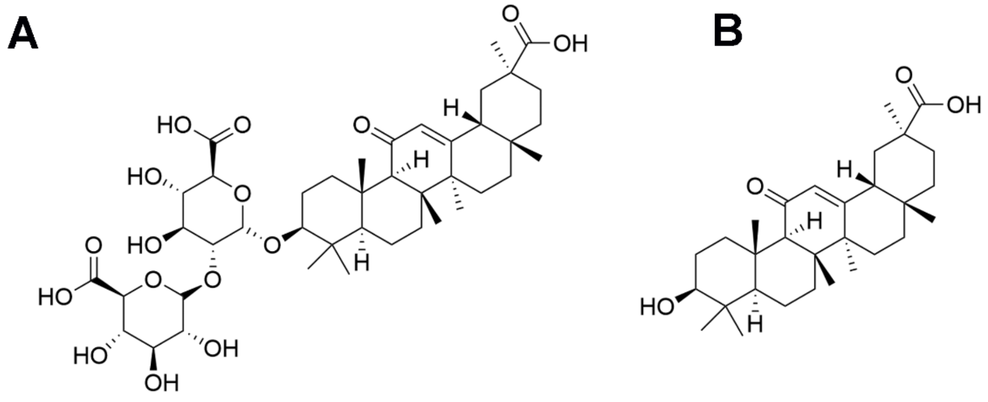

Figure S1. The structure of glycyrrhizin (A) and glycyrrhetinic acid (B).

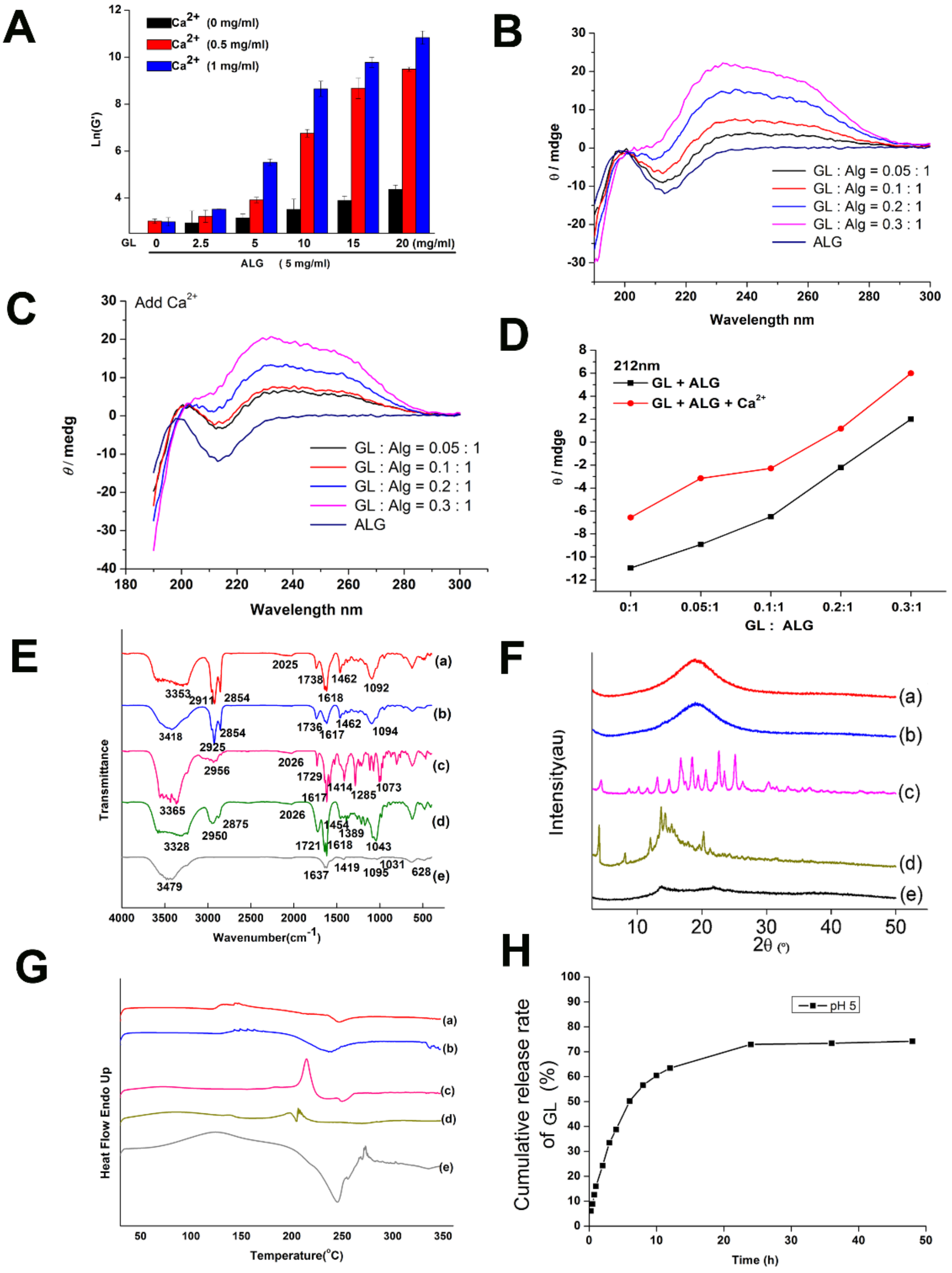

Figure S2 The characterization of DOX/GL-ALG NGPs. The different storage modulus G' values (A). CD value of alginate with adding GL (B). CD value of alginate with adding GL and Ca^{2+} (C). CD value at wavelength 212 nm (D). FT-IR spectra (E); XRD patterns (F); DSC thermograms (G); (a) DOX/GL-ALG NGPs; (b) GL-ALG NGPs; (c) DOX; (d) GL; (e) SA. The release of GL in GL-ALG NGPs (H).

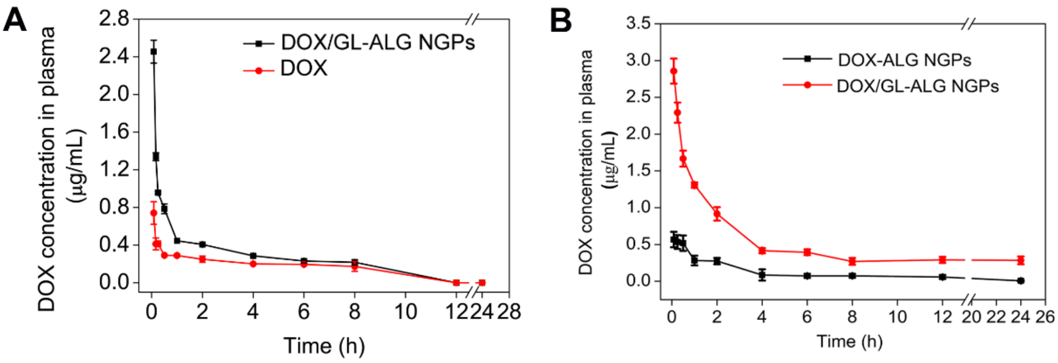

Figure S3. **Pharmacokinetic profiles in rats after i.v. administration of DOX solution, DOX-ALG NGPs and DOX/GL-ALG NGPs at a dose of 2.5 mg DOX-equiv./kg (n = 4).** (Data were given as mean ± SE, n=4).

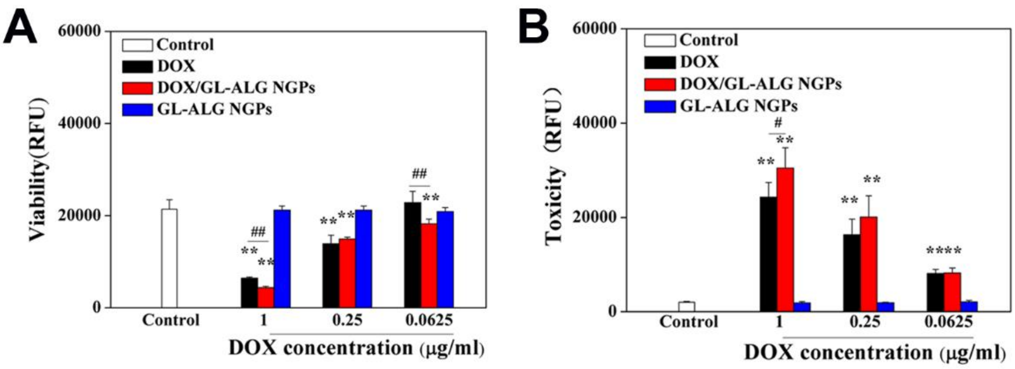

Figure S4. **Cell viability and toxicity of DOX/GL-ALG NGPs on hepatocellular carcinoma cell (HepG2).** $^{**}p < 0.01$, compared with control group, $^{\#\#}p < 0.01$, compared with free DOX group.

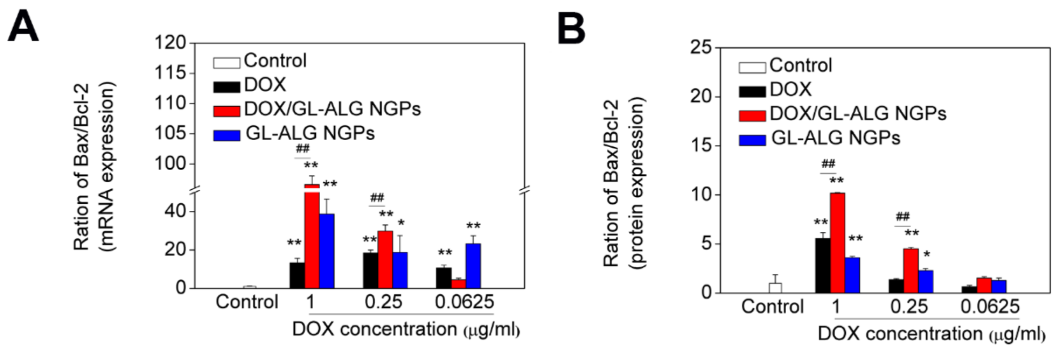

Figure S5. **The mRNA and protein expression ratio of Bax and Bcl-2 *in vitro*.** $^{**}p <$ 0.01, compared with control group, $^{\#\#}p < 0.01$, compared with free DOX group.

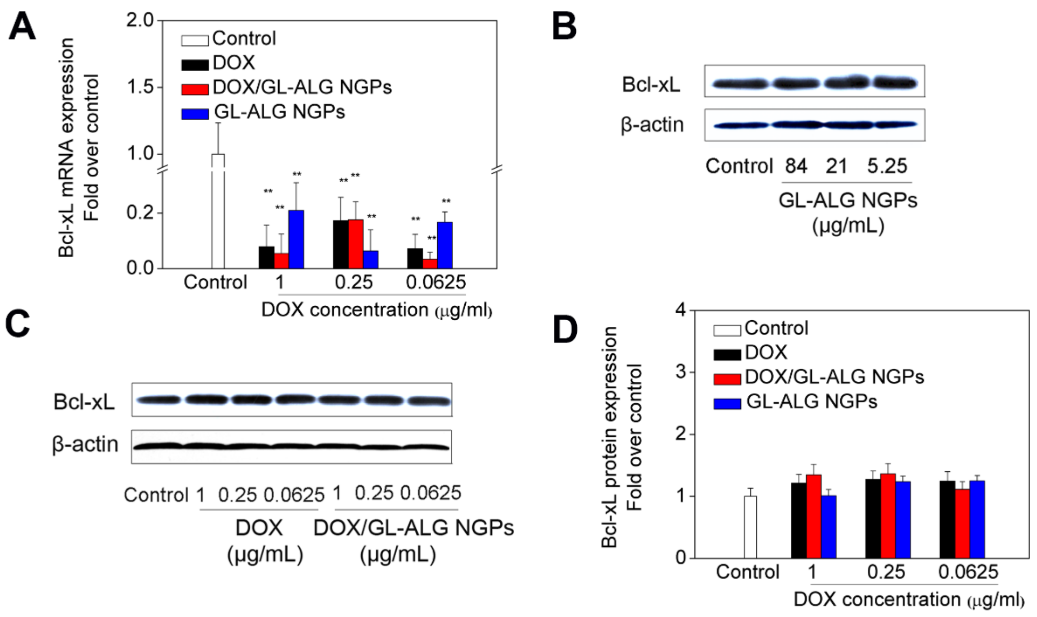

Figure S6. The effect of DOX/GL-ALG NGPs on the mRNA and protein expression of Bcl-xL. The mRNA expression of Bcl-xL (A) was detected by Real-time RT-PCR. The protein expression of Bcl-xL treating with GL-ALG NGPs (B) and DOX, DOX/ GL-ALG NGPs (C) was analyzed by Western Blot. The western blot film was scanned and the intensity (D) was quantified by Image J version 1.51n and normalized to the corresponding β-actin intensity and the controls. Values are means ± S.D. (n=3) from three independent experiments. $^{**}p < 0.01$, compared with control group.

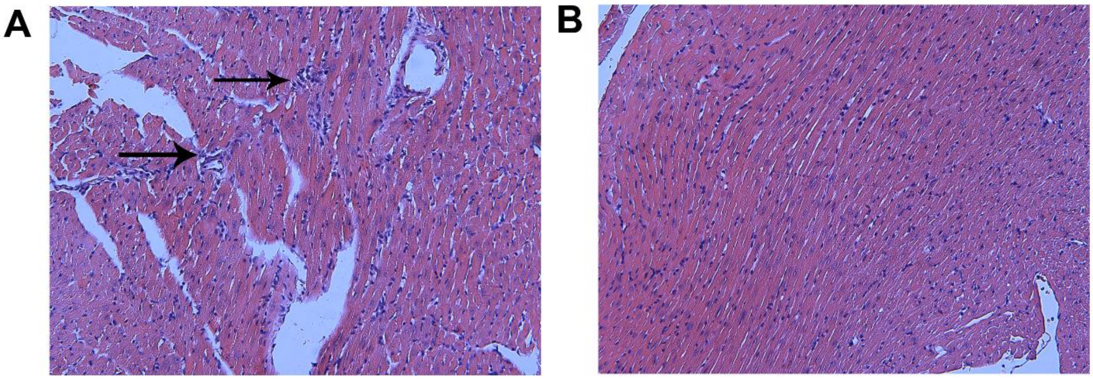

Figure S7. **The myocardial protection of GL on DOX induced cardiotoxicity in mice**. Sections of liver taken from mice received intravenous injections of DOX (4 mg/kg) (A), and co-delivering soluble GL along with DOX (4 mg/kg) (B). (Black arrows: inflammatory cell infiltration)

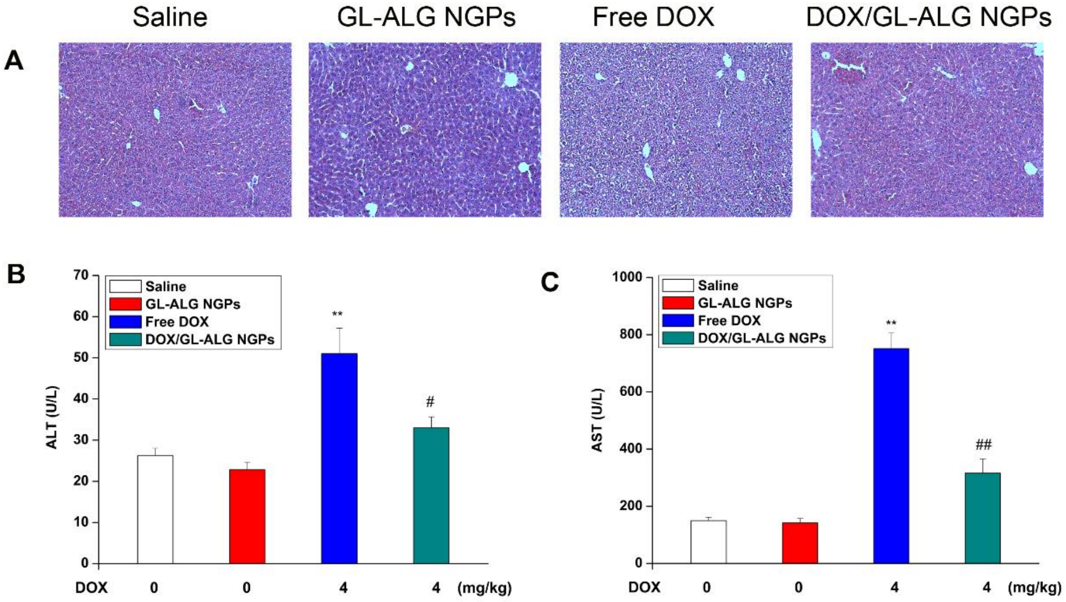

Figure S8. **The effect of DOX/GL-ALG NGPs on hepatotoxicity, AST and ALT levels in mice.** Sections of liver taken from animal received intravenous injections of saline, GL-ALG NGPs, DOX (4 mg/kg) or DOX/GL-ALG NGPs (4 mg/kg) (A). The liver enzyme levels of ALT (B) and AST (C) in mice plasma.

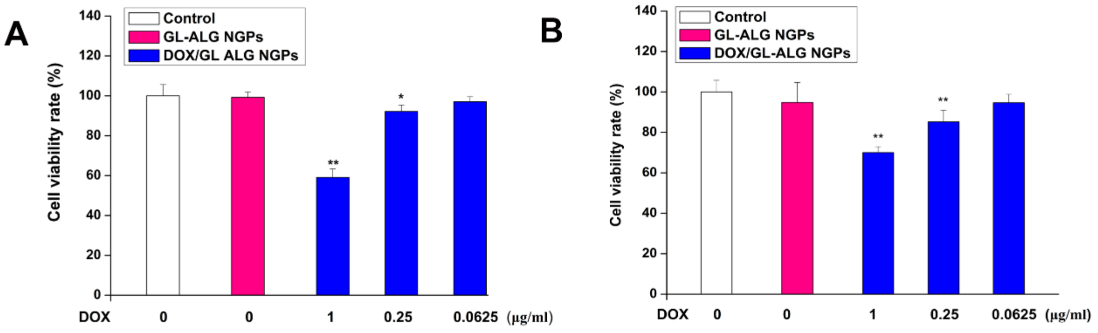

Figure S9. **Cell viability of DOX/GL-ALG NGPs on RAW264.7 cells (A) and L929 cells (B).** $^{**}p < 0.01$, compared with control group.
